# Inhibitory Effects of *Marrubium vulgare* L. Extract on the Female Hormones Based on Bioautography-HPTLC-MS

**DOI:** 10.5812/ijpr-148259

**Published:** 2024-09-15

**Authors:** Sahar Hassannejad, Abdulghany O. I. Sarmamy, Fateme Mirzajani

**Affiliations:** 1 Department of Biology, Salahaddin University-Erbil, Erbil, Iraq; 2 Protein Research Center, Shahid Beheshti University, Tehran, Iran; 3 Knowledge University, Erbil, Iraq

**Keywords:** *Marrubium vulgare *L., Estrogen, Hormones Involved in Women's Diseases, Progesterone, Estradiol, Testosterone, HPTLC

## Abstract

**Background:**

Hormonal imbalances related to women's health, physical activity, and fluctuations are prevalent metabolic disorders in several nations and have significantly impacted women's health for an extended period. The application of individual or combined botanical extracts in traditional, alternative, and complementary medicine is employed to manage and alleviate these issues.

**Objectives:**

The objective of this study is to examine the suppressive properties of horehound (*Marrubium vulgare *L.) on pivotal hormones associated with feminine disorders.

**Methods:**

The horehound plant was exposed to ultrasonic radiation while five different solvents (methanol, ethyl acetate, n-hexane, acetone, and water) were used to extract its components. The individuals were isolated using high-performance thin-layer chromatography (HPTLC). The most powerful compounds were analyzed using a direct antioxidant assay (DPPH test) and a hormone inhibitory assay (Oestrogen, Progesterone, Estradiol, and Testosterone) on the HPTLC plate. The compounds that had a significant effect were then identified using LC-ESI/MSMS.

**Results:**

The antioxidant properties of the extracts and hormone inhibitors were evaluated, and the substances were separated from the HPTLC plate and analyzed using mass spectrometry. The results showed strong antioxidant capabilities, with an IC50 range of 8.24 - 12.42 µg/mL. Moreover, the plant extract showed beneficial effects on hormones associated with female health issues.

**Conclusions:**

The extract was subjected to chemical and molecular analysis using the HPTLC technique, followed by LC-ESI/MSMS. The study revealed the presence of vulgarole, marrubiin, and marrubenol chemicals.

## 1. Background

*Marrubium vulgare *L., commonly known as "horehound," is a plant species belonging to the *Lamiaceae* family, native to North Africa, Western Asia, and Southern Europe. Historically, traditional medicine has utilized the blooming aerial parts and aqueous-methanol extracts of this plant to treat stomach problems and coughs, due to its calming and anti-inflammatory properties. The basic features of the plant are determined by its secondary metabolites (1). A total of 54 secondary metabolites have been documented from horehound plants. Among these, marrubiin stands out as the principal bioactive compound isolated in the aerial parts of the horehound. The plant also contains other compounds, such as vitexin, luteolin, and apigenin (2).

The blooming aerial parts of horehound and its aqueous-methanol extracts are commonly used in traditional medicine to treat stomach ailments and coughs due to their calming and anti-inflammatory properties. In addition, some investigations have explored the gastro-protective, anti-hypertensive, analgesic, hypoglycemic, and antispasmodic properties of these substances (3-5). Furthermore, compounds like premarrubenol, premarrubiin, and vulgarol have been isolated from horehound alongside various flavonoids, steroids, terpenoids, tannins, saponins, and essential oils (6, 7).

Some important properties of marrubiin are its low turnover, high stability, and minimal catabolism. These characteristics make it desirable as a therapeutic compound and an economically valuable nutraceutical (8). These features contribute to its healing effects on different body systems, such as reducing pain, protecting the heart, relaxing blood vessels, protecting the digestive system, reducing spasms, boosting the immune system, reducing swelling, mitigating inflammation, and preventing diabetes. Moreover, marrubiin is a promising precursor for potent active compounds, notably marrubiinic acid and marrubenol.

Marrubiinic acid exhibits antinociceptive effects, whereas marrubenol demonstrates vasorelaxant properties (9). Horehound is also recognized for its anti-inflammatory characteristics. The disruption in homeostatic equilibrium between oxidants and antioxidants, induced by free radicals, amplifies oxidative stress mechanisms. Oxidative stress is identified as a primary contributor to aging and various human ailments such as cancer, diabetes, neurodegenerative conditions, and rheumatoid arthritis. Antioxidants are crucial in delaying, preventing, or inhibiting oxidative harm to target molecules (6). The antioxidant properties of horehound methanol extracts were evaluated in vitro using a DPPH (2,2-diphenyl-1-picrylhydrazyl) free radical scavenging assay. The results indicated robust activity, with a half maximal inhibitory concentration (IC50) value ranging from 8.24 to 12.42 µg/mL (10-12). The antioxidant activity of horehound essential oil was also measured using the same method and found to have an IC50 value of 153.84 µg/mL. This is about twice as high as the antioxidant activity of butylated hydroxytoluene (BHT) (13).

The Photochemiluminescence (PLC) assay was employed to assess the antioxidant activity of the compounds in the presence of superoxide anion radicals. The potent antioxidant effects of methanol and acetone horehound extracts (261.41 and 272.90 µmol TE/g, respectively) were determined, attributable to the reactive oxygen species (ROS) generated in the body. Conversely, we observed lower activity when investigating essential oil and isolated marrubium (1).

Besides its anti-inflammatory properties, horehound extract effectively regulates hormones related to menstrual and women's health conditions. Estimates suggest that the population of menopausal women will grow by approximately 47,000 annually, and projections indicate that by 2030, there will be around 1.2 billion menopausal women worldwide (1-3). Eighty percent of these women encounter symptoms associated with the hormonal changes of menopause, including genitourinary symptoms, vasomotor symptoms, and cognitive problems. However, only 25% of them actively pursue medical aid for their grievances (4-6).

Premature ovarian failure (POF), occurring before the age of 40, affects approximately 1% of women. Autoimmune factors or genetic anomalies primarily cause the condition. Additionally, acquired forms of POF may result from treatments like chemotherapy, radiotherapy, or ovariectomy performed for ovarian cancer (4, 7). The most prevalent form of ovarian dysfunction during the reproductive years is polycystic ovary syndrome (PCOS). This illness has important clinical ramifications, presenting with irregular menstrual cycles, excessive levels of male hormones, and the presence of many cysts on the ovaries. Additionally, PCOS is associated with disturbances in glucose (such as insulin resistance and poor glucose tolerance) and lipid metabolism, which are acknowledged as substantial risk factors for cardiovascular disease (2, 8). Women diagnosed with PCOS have a fourfold higher likelihood of developing type 2 diabetes mellitus (DM) and a 2.8-fold increased risk of gestational diabetes mellitus (GDM) compared to those without the condition. Additionally, around 20% of women with PCOS experience the onset of DM before reaching 40 years of age (3, 8).

Polycystic ovary syndrome impacts approximately 7% of women in their reproductive years. However, the wide range of clinical manifestations associated with PCOS likely leads to the underdiagnosis of 75% of these women during medical consultations (9, 10). In obese women, the prevalence of PCOS is even higher, reaching 15 - 30% (2). Mokhtari et al. investigated the levels of luteinizing hormone (LH), follicle-stimulating hormone (FSH), estradiol, and testosterone hormones in female rats with PCOS. Their findings suggest that the alcoholic extract of white horehound enhances hormonal parameters in polycystic ovarian syndrome (11).

## 2. Objectives

In this study, we prepared and separated various *M. vulgare* extracts using the high-performance thin-layer chromatography (HPTLC) technique. Additionally, the inhibitory effects of the separated compounds on oxidants (DPPH) and hormones-estrogen, progesterone, estradiol, and testosterone—associated with female diseases were studied.

## 3. Methods

After extracting a sample, the HPTLC technique was employed to enhance the separation of the elements in the extract. This allowed for the examination of the effects of *M. vulgare* extracts on antioxidant activity and hormone inhibition. Subsequently, the percentage of inhibition displayed by the separated compounds on the plate was assessed, leading to the discovery of compounds that block hormones. The HPTLC plate was used to isolate hormone-inhibitory compounds, which were then analyzed using mass spectrometry.

### 3.1. Plant Material Preparation

Horehound plants were collected in October 2022 from Ali Abad, Zarin Gol, and Siah Rud Bad, Mazandaran Province, Iran. The herbarium at Shahid Beheshti University in Tehran, Iran, conducted the identification and coding of the plant samples. The approved plant underwent three rounds of washing with distilled water (DW) and was then air-dried on a drying bench for a week at room temperature (25°C) in the absence of light. Afterward, it was ground using a benchtop grinder for extraction.

### 3.2. Chemicals

Hormones, including progesterone, estradiol, methanol, chloroform, toluene, ethyl acetate, ethanol, formic acid, acetic acid, estrogen, testosterone, and HPTLC plates (precoated silica gel aluminum plate 60F254) were acquired from Merck Co. (Germany). The DPPH, BHT, Na₂HPO₄, and NaH₂PO₄ were acquired from Sigma Aldrich Co. (Germany).

### 3.3. Phytochemical Extraction

The extraction process was performed using five different solvents: methanol, ethyl acetate, n-hexane, acetone, and water (14). The extraction was conducted for 30 minutes at room temperature (20°C) with 600 W ultrasonic irradiation. Five grams of ground plant powder were added to 100 mL of each extraction solvent. The extract was filtered twice using a 0.45 μm syringe filter, concentrated under a rotary vacuum evaporator, and stored at 30 °C. The extracts were kept at 4°C until further study.

### 3.4. Instruments for Separation

A 100 mL syringe from CAMAG (Switzerland) was used to spot 20 µL of sample solution onto an HPTLC plate in 8 mm wide bands using a sample applicator from CAMAG (Switzerland). Various solvent systems were employed to measure the spots of the plant extracts. The plates were subjected to UV-Vis detection at 254 and 365 nm wavelengths. The optimal mobile phase system for the separation of methanol extract consists of a mixture of acetone, toluene, and acetic acid in a volumetric ratio of 3:17:0.5. The optimal mobile phase system for the separation of horehound water extract is a mixture of acetone, n-hexane, and ethyl acetate in a ratio of acetone:toluene:formic acid (3:17:0.5 v/v). Lastly, the most effective mobile phase system for separating horehound water extract is a mixture of water, methanol, and acetic acid in a ratio of 15:80:5 (v/v).

### 3.5. Anti-oxidant (DPPH) Assay

Each extract's DPPH radical scavenging capacity was assessed using Brand-Williams's method, with certain modifications (15). An antioxidant molecule reduces the radicals, eliminating the maximum absorption of DPPH radicals at a wavelength of 515 nm. A solution of DPPH in methanol (6 × 10⁻⁵ M) was prepared daily. Subsequently, 200 µL of this solution was combined with 100 µL of the plant extract solutions in a 96-well plastic tray. The samples underwent a 20-minute incubation period. The absorbance decrease at 515 nm was measured at a temperature of 25°C and a rotating speed of 450 rpm. The experiment was conducted three times. The equation used to compute the radical scavenging activity is as follows:


$$Inhibition\%=1-\left(\frac{AS-AB)}{AC}\right)\times 100$$

Where AS, AB, and AC represent the absorbance of the samples, the blank absorbance (extract solution), and the absorbance of the control (DPPH solution), respectively (12, 13).

### 3.6. Hormone Activity Assay

Progesterone, estradiol, and testosterone from commercial diagnostic kits prepared by Abcam Co. ELISA Kit (Washington, USA) were used to investigate the interaction of the extracts with estrogen hormones. The experiments were carried out at a temperature of 22 to 28°C. Ten microliters of extract and fifty microliters of hormone standard solution (10 ng/mL) were mixed with fifty microliters of enzyme conjugate solution. The plate was gently shaken for 30 seconds. Then, fifty microliters of conjugated biotin solution were added, and the well was shaken again and incubated for 60 minutes at 37°C. Wells were emptied by either turning them upside down or using aspiration. After 15 minutes, one hundred microliters of 3,3',5,5'-tetramethylbenzidine (TMB) reagent were added. Fifty microliters of stop reaction solution were added, and the plate was gently shaken for 20 seconds until all the blue color turned yellow. Finally, the absorbance of the samples was measured at a wavelength of 625 nm (16-18).

### 3.7. Direct On-plate Autobiography Analysis

To determine the antioxidant DPPH inhibitory assay, the developed HPTLC plate was placed in a DPPH• solution in methanol (6 × 10⁻⁵ M) for 10 minutes in a dark place, then dried at 40°C. The plates turned purple, with yellow and white areas indicating medium and high antioxidant activity, respectively (19).

For hormone inhibitory activity, the developed HPTLC plate was placed in a reaction solution (containing hormone standards at 0.05 mM, enzyme conjugate at 0.01 mM, and conjugated biotin at 0.2 mM) at 37°C for 20 minutes. The plate was then incubated for 5 minutes at 37°C. The TMB reagent solution was sprayed on the plate. The TMB sublimation process revealed yellow spots on the plate, indicating the presence of hormone-inhibitory action. The plates exhibited blue coloration, while the yellow regions suggested the presence of hormone inhibitors.

### 3.8. Liquid Chromatography-Mass Spectrometry Identification

For the mass spectrometry experiment, 300 μL of the extract, specifically the methanolic extract that blocked hormones most effectively, was applied to a 20 × 10 cm preparation plate. The plate was then subjected to optimal separation conditions. Upon completion of the separation process, the relevant spot was removed from the plate and dissolved in a methanol-water mixture with a ratio of 30:70. After thoroughly mixing for 1 minute, the mixture was centrifuged for 10 minutes at 10,000 rpm. The resultant liquid was used for injection into LC/MS. A Dionex LCP model HPLC system with an ultimate binary pump and a C18 Dionex PreMap precondition column measuring 300 μm × 5 mm was used for chromatographic analysis. An autosampler equipped with a 5 μL loop and an injection volume of 1/2 operated at a temperature of 35°C.

A Dionex Co. PerpMap100 column made of fused silica C18 capillary (3 μm, 100 Å, 75 μm × 150 mm, connected to PRP-X100 C18 Guard Columns) was also part of the chromatography setup (California, USA). The mobile phase consisted of two components: eluent A, comprising a mixture of water with 1.2% acetonitrile and 0.08% trifluoroacetic acid, and eluent B, consisting of acetonitrile with 32% water and 0.12% trifluoroacetic acid. After 5 minutes, the washing process began with eluent A, followed by a transition to 25% eluent B. Subsequently, over 5 minutes, the concentration of eluent B increased to 100%, maintaining this composition for a total of 10 minutes for washing. Then, eluent A was gradually increased to 100% over 5 minutes, and the system underwent a 10-minute wash using this eluent. The flow rate was maintained at 150 nL/min, and the separation lasted for 30 minutes, monitored via UV detection at 370 nm. The study utilized an LC-ESI-MS system, incorporating a PicoTip^®^ emitter sourced from New Objective (MA, USA) as the spray nozzle. The Thermo Fisher Scientific Finigan LTQFT Ultra mass spectrometer (Germany) featured an NESI ion generator. It employed Nano Electrospray Ionization, along with both collision induced dissociation (CID) and electron capture dissociation (ECD) methods for ion fragmentation. The setup included an Ion Trap (IT) mass detector with a full width at half maximum (FWHM) of 100 Da and an accuracy exceeding 0.5 ppm. This equipment was connected to HPLC for metabolite identification. The Xcalibur software oversaw instrument control, data collection, and processing. For ESI-MS in negative mode, a capillary voltage of 2.0 kV and a skimmer cone voltage of 20 V were typically employed.

## 4. Results

**Figure 1.**
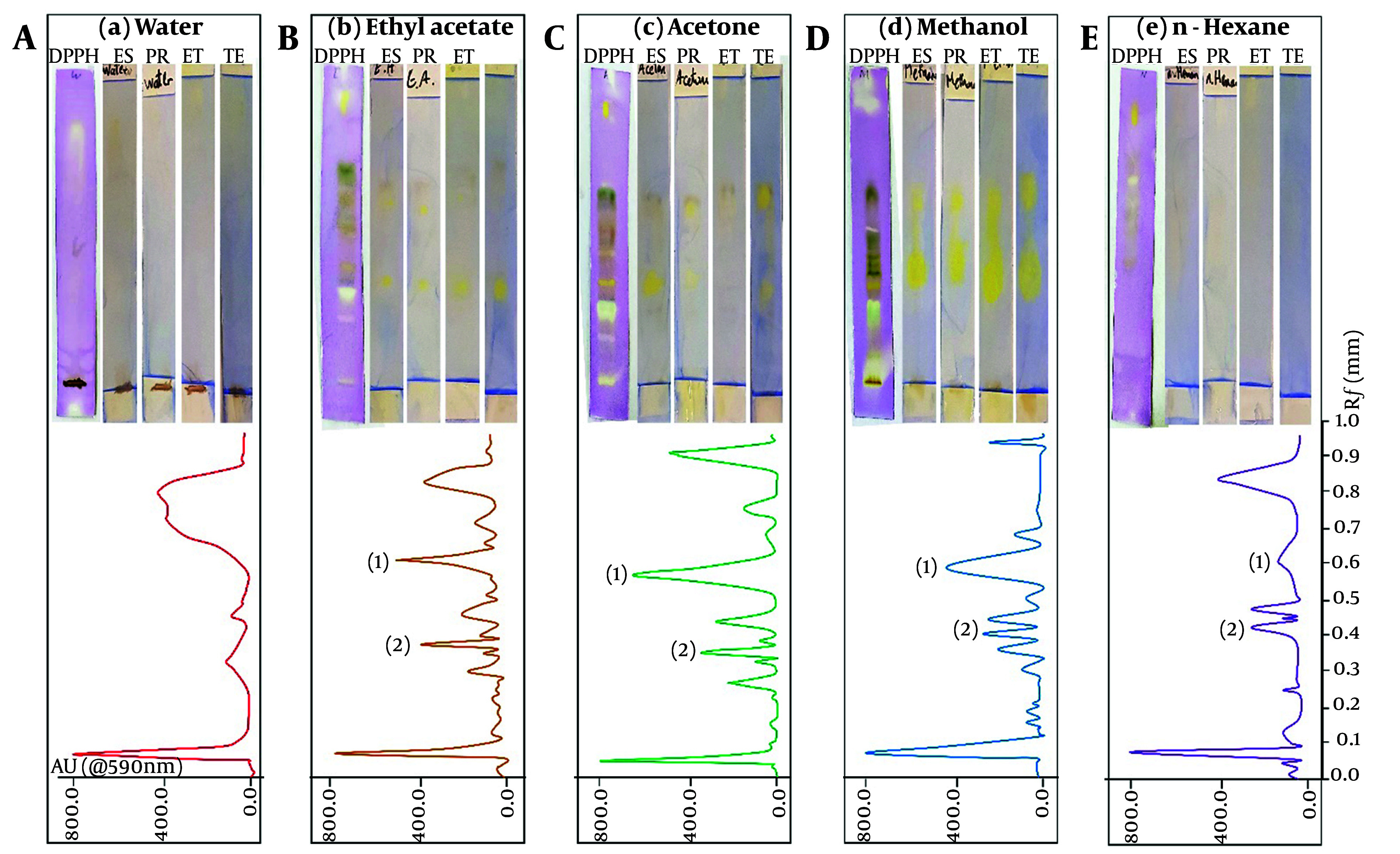
Bio-autography under DPPH and hormonal (Es: Estrogen, Pr: Progesterone, Et: Estradiol and Te: Testosterone) inhibition (upper) and HPTLC separation (bottom) of *Marrubium vulgare* L., methanolic, acetone, ethylacetate, aqueous, and hexane extracts. Note: Areas No. 1 and 2 were selected for mass spectrometry analysis.

After preparing methanol, acetone, ethyl acetate, aqueous, and hexane extracts, the antioxidant effect was evaluated using the DPPH test, and the degree of direct inhibition of the studied hormones was recorded (Table 1). Butylated hydroxytoluene was used as a positive control for antioxidant activity (20). All experiments were analyzed in triplicate. As shown in Table 1, the highest antioxidant activity and enzyme inhibition were observed in the methanolic, acetone, and ethyl acetate extracts, respectively. The aqueous and hexane extracts exhibited minimal activity, which could not be reported in some cases (hexane extract). Subsequently, all the extracts were separated using the HPTLC method. For this, the extracts were applied to HPTLC plates and subjected to various washing systems to determine the most effective solvent system for optimal separation. Figure 1 shows the separation spectrum of each extract at 360 nm and the optimal separation mobile phase systems. These plates were then evaluated directly (bio-autography) according to the reported method to study the antioxidant effect and enzyme inhibition (Figure 1). 

**Table 1.** DPPH and Direct Hormone Inhibition of *Marrubium vulgare*

Extract Type	IC50 DPPH ^a^	Hormone Inhibition (%)			
		Estrogen	Progesterone	Estradiol	Testosterone
**Water**	112. 06 ± 0.0052	5.31 ± 0.0003	3.25 ± 0.0041	3.08 ± 0.0027	2.01 ± 0.0028
**Ethyl acetate**	88.61 ± 0.0024	15.93 ± 0.0025	13.24 ± 0.0011	15.12 ± 0.0031	14.25 ± 0.0024
**Acetone**	53.44 ± 0.0081	22.18 ± 0.0013	18.12 ± 0.0023	16.81 ± 0.0018	19.24 ± 0.0009
**Methanol**	31.89 ± 0.0033	62.51 ± 0.0038	53.22 ± 0.0018	79.33 ± 0.0032	72.36 ± 0.0015
**n-Hexane**	215.22 ± 0.0062	5.31 ± 0.0003	trace	trace	2.01 ± 0.0028

^a^ Against BHT as positive control (IC50 22.78 ± 0.0021 g/g).

**Figure 2.**
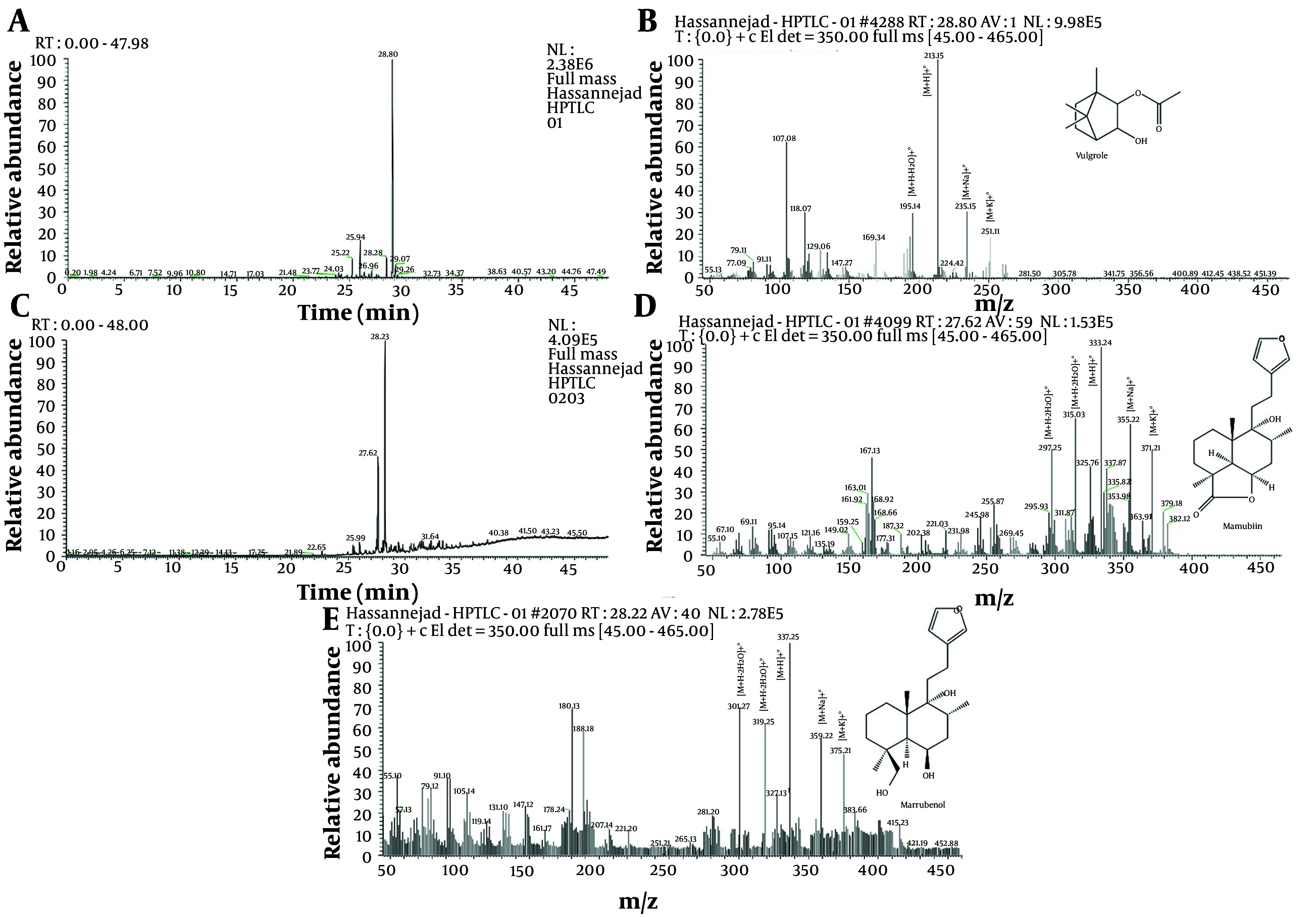
Liquid chromatography was conducted on selected areas 1 (A) and 2 (C) using HPTLC chromatography, along with their related processes. Mass spectrometry (spectrum B related to separation A and spectrums D and E related to separation C).

Based on the comparison of bio-autography results and the separation spectrum, two areas, numbered 1 and 2, were selected on the plates to check the constituent molecules. The HPTLC bands were introduced into a liquid chromatography mass spectrometry (LC-MS) system for mass spectrometry analysis. Following this, a study was conducted to evaluate the degree of separation and the characteristics of the separated substances. Figure 2 shows the high-performance liquid chromatography (HPLC) results for the targeted area of the methanolic extract (Figures 2A and C). It also displays the mass spectrometry results for the three distinct peaks observed in chromatograms 2A and 2C (Figure 2B, D, and E). The HPTLC plate reveals a molecule with dual antioxidant and hormone inhibition properties. This compound comprises three components with relatively close retention times. Liquid chromatography separated these components, revealing retention times of 28.80 minutes in the first region and 27.62 and 28.23 minutes in the second region. Their mass spectrometric spectra were subsequently acquired.

The mass spectra (MS) of both substances display a similar pattern, although the observed peak intensities vary (21). Adduct ions were used to identify the compounds. Among the prominent adduct ion peaks identified are [M+Na]+•, [M+K]+•, [M+H]+•, [M+H-H_2_O]+•, and [M+H-3H_2_O]+•. These ions and fragments were identified based on a study from the Metlin Online Library.3,32 - 35 The adduct peaks identified for each molecule are displayed on the spectrum and compared with available reference data. For Vulgarole, the positive adducts identified are 195.14, 213.15, 235.15, and 251.11 Da. Marrubiin was identified using positive adducts of 297.25, 315.03, 325.76, 355.22, and 371.21 Da. Finally, Marrubenol was identified using positive adducts of 301.27, 319.25, 337.25, 359.22, and 375.21 Da (5, 22, 23).

Based on the mass spectrometry analysis and recommendations from the software, three compounds were discerned: Vulgarole, marrubiin, and marrubenol. The mass spectrum indicates that all identified adducts are present in each compound.

## 5. Discussion

The anti-inflammatory properties of *M. vulgare* extract proved crucial in its protective role (24, 25). Due to its high content of diterpenes and polyphenols, it can serve as a powerful antioxidant and anti-inflammatory treatment for stress-induced diseases (26). Reactive oxygen species (ROS), which include oxygen radicals and their reaction products with biological molecules, can be harmful to cells and tissues. Antioxidant activity is a complex mechanism that often involves multiple pathways. Testing for antioxidant activity in pure compounds or extracts is challenging and requires various techniques (17).

In this study, the highest antioxidant activity was observed in the methanolic, acetone, and ethyl acetate extracts. Research suggests that 200 mg/kg of methanolic extract significantly reduces inflammation. The anti-inflammatory effects of a hydromethanolic extract of horehound aerial parts have also been studied (27). The methanolic extract of horehound leaves demonstrated strong antioxidant properties, with an IC50 of 35 µg/mL for DPPH and 25 µg/mL for 2,2′-azinobis-(3-ethylbenzothiazoline-6-sulfonic acid) tests. Additionally, we evaluated the antioxidant activity of horehound leaf extracts in rapeseed (Brassica napus L.) oil at a temperature of 80°C (28).

In this study, we assessed the effectiveness of the extracts using peroxide value, weight gain, and UV absorption measurements. The acetone extract demonstrated superior antioxidant activity compared to the water extract. The total antioxidant activity of horehound aqueous extracts was measured in a separate study (29) using a two-stage Trolox-based test (30). The results indicated that the aqueous extracts had an antioxidant potential of 560 µmol/g Trolox equivalent/g dry weight. Results obtained by isolating compounds on the HPTLC plate with antioxidant and hormone inhibition actions show that the extracts in this study contain three compounds with highly similar retention times. We separated the compounds using liquid chromatography and obtained mass spectrometric spectra. In the first region, HPLC retention times ranged from 28.80 to 28.23 minutes, and in the second region, they ranged from 27.62 to 28.23 minutes. The MS of the two drugs have a similar overall shape, although the peak frequencies differ. The most prominent adduct ion peaks are [M+Na]+•, [M+K]+•, [M+H]+•, [M+H-H_2_O]+•, and [M+H-2H_2_O]+•.

Similar studies show that horehound contains up to 3 mg/g of fresh weight (31, 32) of labdane-type diterpenes, with marrubiin (0.12 - 1%) being the most abundant, followed by its precursor pre-marrubiin (0.13%), 12(S)-hydroxymarrubiin, 11-oxomarrubiin, marrubenol, and marruliba-acetal (33). The diterpenoid fraction also includes vulgarole A, deacetylvitexilactone, carnosol, and deacetylforskolin (33, 34). Horehound's methanol extract has an estimated marrubiin concentration of 156 mg/g (28). The dry flowering stalk can irritate the mucosa and is used by herbalists to treat cramps and irregular menstruation. Flavonoids such as apigenin can lower LH secretion by competitively inhibiting flunitrazepam binding (35). Studies have shown that testosterone in the horehound extract lowers LH levels. Beta-sitosterol inhibits testosterone production by reducing cholesterol levels (36). Beta-sitosterol also lowers estradiol levels by inhibiting aromatase enzyme activity, thus blocking the conversion of testosterone to estrogen. Apigenin and ursolic acid, found in white horehound extract, inhibit cytochrome P450 and prevent the production of steroid hormones like progesterone (37-40).

### 5.1. Conclusions

Our research on the aerial portions of *M. vulgare* L. emphasized how the choice of solvent for extraction affects the chemical composition of the plant extract. Quality indicators included direct suppression of hormone production and antioxidant activity. The methanolic extract showed the highest effectiveness. The IC50 of the methanolic extract against DPPH was 31.89 ± 0.0033. The bioautography HPTLC analysis for hormone inhibition percentage (Estrogen 62.51 ± 0.0038, Progesterone 53.22 ± 0.0018, Estradiol 79.33 ± 0.0032, and Testosterone 72.36 ± 0.0015) helped identify effective compounds. Mass spectrometry revealed the most reactive compounds to be marrubiin, marrubinol, and vulgarole. These are among the terpenoid chemicals identified through quantitative analysis. Previous studies have highlighted the valuable medical uses of marrubiin, marrubinol, and vulgarole, noting their potential as natural antioxidants for treating female diseases associated with hormonal imbalances. However, further research is needed, as existing studies have been insufficient and sometimes conflicting.

## Data Availability

The processed data used and analyzed during the current study are available from the corresponding author upon reasonable request.
